# The unwanted cadmium: Uncovering the genetic factors for cadmium accumulation in wheat

**DOI:** 10.1093/plphys/kiae360

**Published:** 2024-06-27

**Authors:** Munkhtsetseg Tsednee

**Affiliations:** Assistant Features Editor, Plant Physiology, American Society of Plant Biologists; Agricultural Biotechnology Research Center, Academia Sinica, Taipei 11529, Taiwan

Cadmium (Cd) is a nonbeneficial heavy metal that is toxic to living organisms. Nevertheless, it enters our food chain. Common staple cereals, such as wheat and rice, accumulate Cd in their grains as a “hidden toxicant” and can cause organ damage in humans (Clemens et al. 2013). Although Cd is naturally present in trace quantities, its contamination in agricultural farmland caused by mining, industrial emissions, and fertilizer provides abundant Cd available for uptake by crops (Liang et al. 2024). Most plants do not have specialized transporters for Cd. Instead, because it is present as a divalent cation, it gets into roots through other transporters, for example, those optimized for iron (Fe^2+^) or manganese (Mn^2+^) ions (Huang et al. 2024).

Wheat is an important human food crop, in high demand and with increasing consumption in nearly 90 countries. Globally, it provides about 20% of human dietary energy (Shewry and Hey 2015). Therefore, lowering Cd content in wheat is critical for ensuring food safety and human health.

Cd accumulation in wheat grain, as in other cereals, is a complex trait governed by its transport routes within plant tissues, including root Cd uptake, root-to-shoot translocation, and shoot redistribution (Huang et al. 2024). In addition, subcellular Cd compartmentation and sequestration also impact Cd accumulation in grain (Ning et al. 2023). Uncovering genetic factors controlling Cd transport and contents in wheat provides valuable strategies for developing “low-Cd-wheat.”

In this issue of *Plant Physiology*, Cheng et al. (2024) used a quantitative trait loci (QTL) mapping approach and identified genetic architecture associated with Cd accumulation in Polish wheat (*Triticum polonicum* L.). First, to quantify Cd across different tissues of the plant, the authors measured Cd concentrations in 12 distinct tissues of 198 recombinant inbred lines (RIL_DT) and their parents, dwarf (DPW) and tall (TPW) Polish wheat containing low and high concentrations of Cd in their grains, respectively. They further identified 34 major QTLs, including 29 novel QTLs, associated with grain and tissue Cd concentrations. Six novel QTLs for grain Cd concentration explained 8% to 17% of the phenotypic variation.

Using Cd concentrations in tissues, the authors next calculated Cd uptake, translocation, and distribution in wheat lines and detected 14 novel QTLs linked to these traits. One QTL for root-to-shoot Cd translocation explains 21% to 32% of the phenotypic variation. Moreover, several QTLs showed pleiotropic coregulations for multiple traits, e.g. 5 QTLs for grain Cd concentration and Cd uptake; 1 QTL for root-to-shoot Cd translocation and Cd concentrations at different tissues. Further validation of the novel QTLs by comparing them with the physical positions of Cd-related genes in durum and common wheat suggested that the genetic factors for Cd stress in Polish wheat might be different.

**Figure 1.**
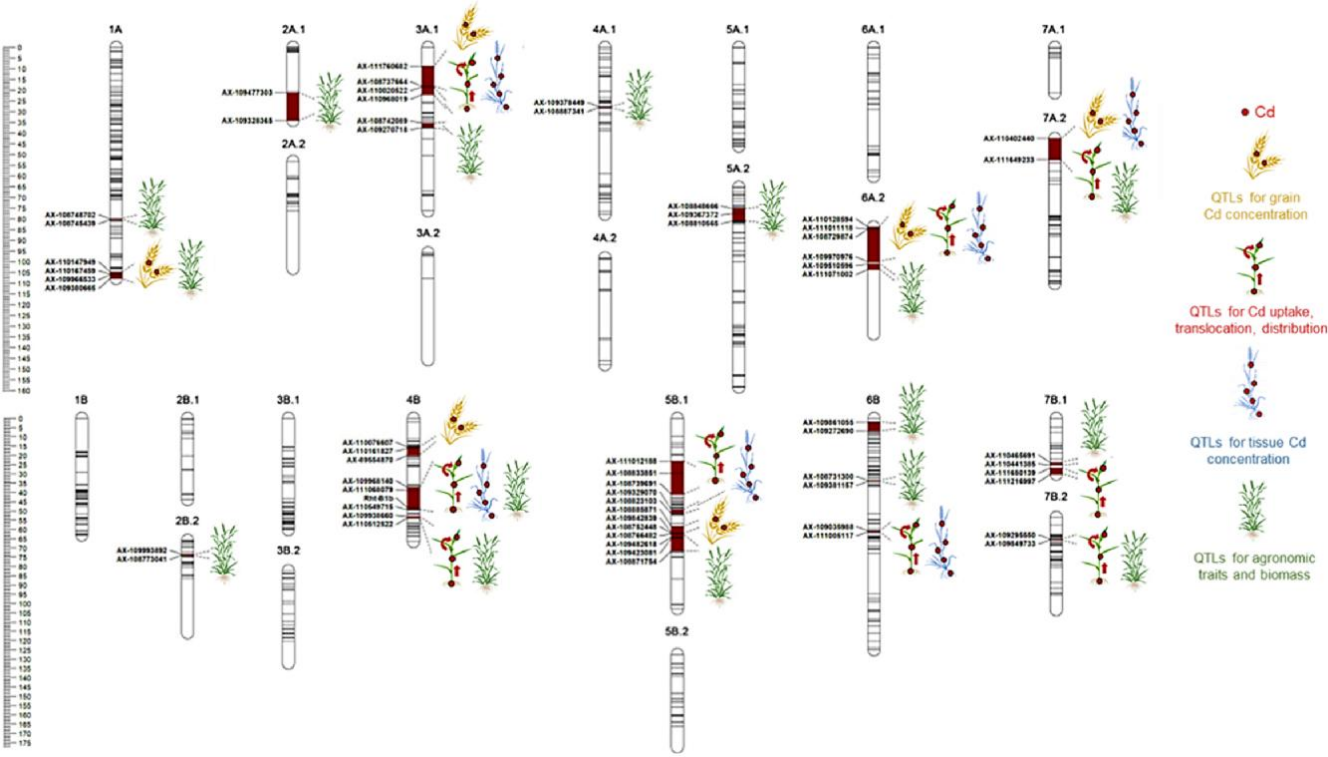
Graphical display of chromosome positions of QTLs. Location of QTLs associated with Cd concentration, uptake, translocation and distribution, agronomic traits, and tissue biomass. The text on the left of each chromosome are the SNP markers. The red patterns on chromosome bars represent the location of organ-specific or pleiotropic QTLs. The simplified illustrations on the right of each chromosome represent the QTLs (modified from Cheng et al. (2024)).

Cd accumulation in plants reduces their growth and development (Yu et al. 2024). Therefore, the authors also performed QTL mapping for 8 agronomic traits and detected 27 major QTLs. Indeed, most of them overlapped with the QTLs for Cd uptake, translocation, distribution, and tissue Cd concentrations. Altogether, Cheng et al. (2024) identified 70 QTLs—57 novel QTLs, 61 QTLs at 14 pleiotropic loci, and 9 QTLs at organ-specific loci—associated with Cd and agronomic-related 25 traits in Polish wheat (Fig. 1).

Furthermore, based on fine-mapping and annotated gene expression analysis in DPW and TPW tissues combined with their homologs involved in Cd stress in other plants, the authors identified potential candidate genes on 7 different chromosomes. The candidates include transporter and ATPase genes such as *Zinc transporter 4* (*ZIP4*), *Cation/calcium exchanger 2* (*CCX2*), *Metal tolerance protein 4* (*MTP4*), and *Plasma membrane ATPase 1* and *6* (*PMA1* and *PMA6*).

One of the novel QTL regions on chromosome 4B that affects grain Cd concentration (named *QGr_Cd_Conc-4B*) includes only a single candidate gene putatively encoding a CCX2 transporter, *TpCCX2-4B*, which is primarily expressed in roots and grains. Differences in the promoter sequence of this gene classify the recombinant inbred lines and natural wheat populations into 2 groups: DPW-derived *TpCCX2-4B^DPW^* and TPW-derived *TpCCX2-4B^TPW^* genotypes. Interestingly, grain Cd concentrations in these 2 groups appear distinct, being 16% to 24% lower in *TpCCX2-4B^DPW^* genotypes than in *TpCCX2-4B^TPW^* genotypes. These results prompted the authors to further validate *TpCCX2-4B* functions as a candidate gene for *QGr_Cd_Conc-4B*.

At the grain-filling stage, *TpCCX2-4B* mRNA accumulation is higher in DPW grains than in TPW grains, possibly as a consequence of differences in their promoter sequences. The higher mRNA accumulation in DPW grains correlates with lower Cd accumulation in the grain. The TpCCX2-4B protein is located at both the endoplasmic reticulum and plasma membrane and, based on a yeast transport assay, acts as a Cd efflux transporter (Cheng et al. 2024). To examine its function in plants, the authors generated *TpCCX2-4B* overexpressing lines in rice and observed that the overexpression of this gene results in significantly lower Cd accumulation in rice grains, consistent with the results from wheat. Additionally, *TpCCX2-4B* overexpressing rice accumulates lower Cd content in their lower nodes, lower internodes, lower leaves, node I, and roots without affecting Cd levels in other tissues and Cd translocation and distribution. These results suggest that overexpression of *TpCCX2-4B^DPW^* inhibited Cd uptake and confirms *TpCCX2-4B* as a potential candidate gene for *QGr_Cd_Conc-4B* and for lowering the grain Cd concentrations.

Overall, the many novel QTLs identified by Cheng et al. (2024) will provide important resources and targets for further genetic improvement of “low-Cd-wheat” cultivars. Moreover, the candidate gene *TpCCX2-4B* directs to a new, yet-to-be-explored avenue for successful controlling of Cd accumulation in wheat grain.
